# Supplementary material for: The Construction and Exploration of a Comprehensive MicroRNA Centered Regulatory Network in Foxtail Millet (Setaria italica L.)
Source: Front Plant Sci. 2022 May 6;13:848474. doi: 10.3389/fpls.2022.848474 (PMC9121102; doi:10.3389/fpls.2022.848474)
Supplement: Supplementary file 1 [file Data_Sheet_1.zip › Sit-miRN3026b_Seita.9G193000.1_962_TPlot.pdf]

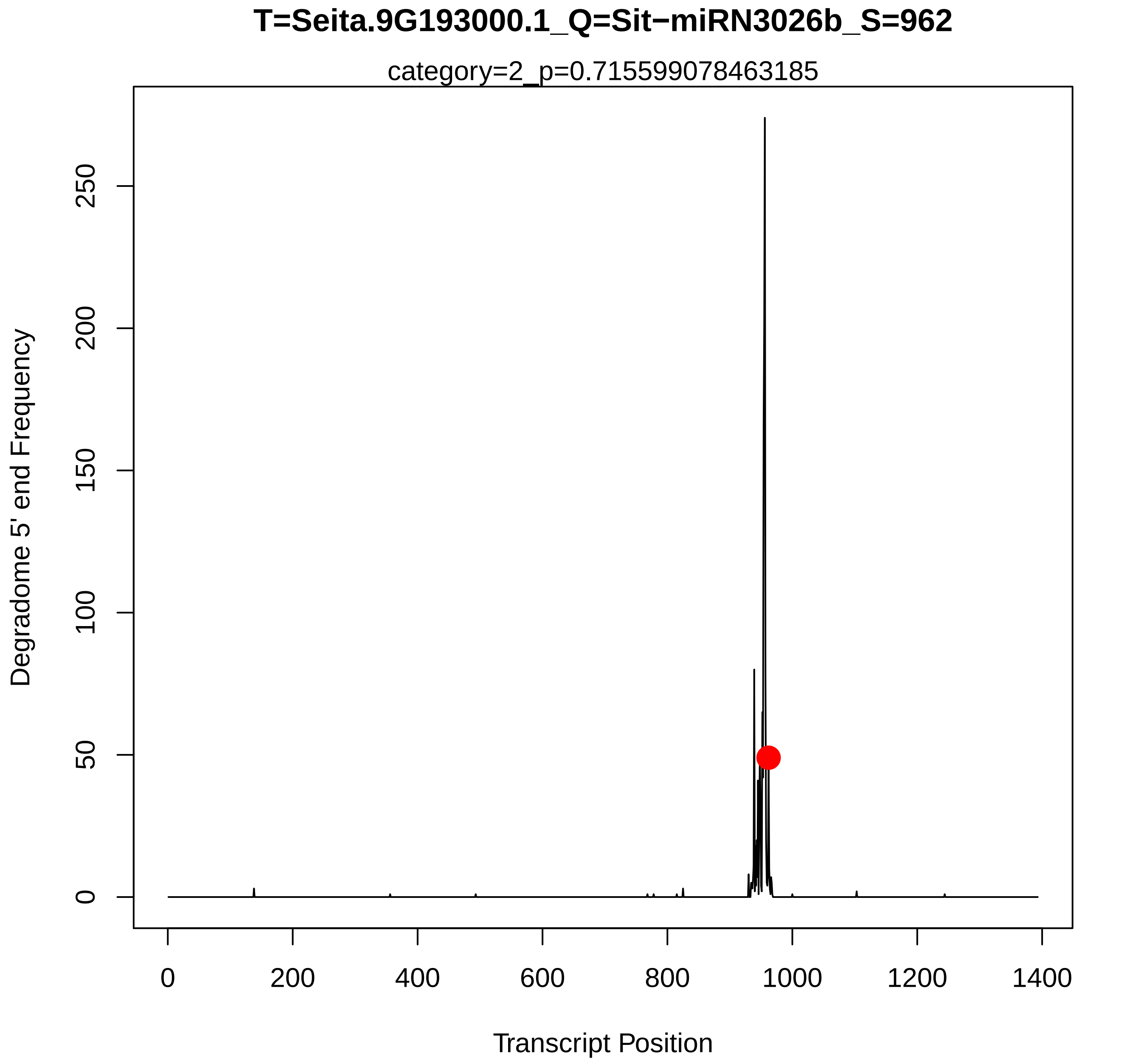
T=Seita.9G193000.1_Q=Sit−miRN3026b_S=962
category=2_p=0.715599078463185
Degradome 5' end Frequency
0
50
100
150
200
250
0
200
400
600
800
1000
1200
1400
Transcript Position
